# Prenatal alcohol exposure results in brain region- and sex-specific changes in *circHomer1* expression in adult mouse brain

**DOI:** 10.3389/fnins.2023.1087950

**Published:** 2023-02-17

**Authors:** Grigorios Papageorgiou, Stephen K. Amoah, Caroline Pierotti, Madison Otero, Sophie Eckel, Kacie Coffey, Andrea M. Allan, Kevin K. Caldwell, Nikolaos Mellios

**Affiliations:** ^1^Department of Neurosciences, University of New Mexico School of Medicine, Albuquerque, NM, United States; ^2^Autophagy, Inflammation, and Metabolism (AIM) Center, Albuquerque, NM, United States

**Keywords:** circRNA, lncRNA, *H19*, *circHomer1*, prenatal alcohol (ethanol) exposure, adult brain

## Abstract

Circular RNAs (circRNAs) are a novel category of covalently-closed non-coding RNAs mainly derived from the back-splicing of exons or introns of protein-coding genes. In addition to their inherent high overall stability, circRNAs, have been shown to have strong functional effects on gene expression *via* a multitude of transcriptional and post-transcriptional mechanisms. Furthermore, circRNAs, appear to be particularly enriched in the brain and able to influence both prenatal development and postnatal brain function. However, little is known about the potential involvement of circRNAs in the long term influence of prenatal alcohol exposure (PAE) in the brain and their relevance for Fetal Alcohol Spectrum Disorders (FASD). Using circRNA-specific quantification, we have found that *circHomer1*, an activity-dependent circRNA derived from Homer protein homolog 1 (*Homer1*) and enriched in postnatal brain, is significantly down-regulated in the male frontal cortex and hippocampus of mice subjected to modest PAE. Our data further suggest that the expression of *H19*, an imprinted embryonic brain-enriched long non-coding RNA (lncRNA), is significantly up-regulated in the frontal cortex of male PAE mice. Furthermore, we show opposing changes in the developmental- and brain region specific- expression of *circHomer1* and *H19*. Lastly, we show that knockdown of *H19* results in robust increases in *circHomer1* but not linear *HOMER1* mRNA expression in human glioblastoma cell lines. Taken together, our work uncovers notable sex- and brain region-specific alterations in circRNA and lncRNA expression following PAE and introduces novel mechanistic insights with potential relevance to FASD.

## Introduction

Fetal alcohol spectrum disorders (FASD) are an umbrella of alcohol-related neurodevelopmental disorders characterized by both growth, neurocognitive, and neurobehavioral deficits (Carter et al., 2016; Marquardt et al., 2020). FASD is characterized by various neurodevelopmental abnormalities that could lead to long-lasting neuropsychiatric impairments and are suggested to be dependent on the dose, timing, and duration of prenatal alcohol exposure (PAE) (Chudley et al., 2005; Valenzuela et al., 2012). Noteworthy in the USA almost $5.4 billion per year is spent on treating FASDs (Popova et al., 2011), which impose a serious socioeconomic burden to patients and their families. It has been proposed that PAE could affect the regulation of gene expression by either impacting DNA methylation and histone modifications or by altering the expression of non-coding RNAs and especially microRNAs (Kaminen-Ahola, 2020). Previous studies have portrayed the miRNA dysregulation following ethanol exposure in CNS (Sathyan et al., 2007; Pappalardo-Carter et al., 2013; Mahnke et al., 2019), primarily focusing on miRNAs that are significant in the regulation of neurodevelopmental processes and inflammatory gene expression (Ibáñez et al., 2020). Such studies build up the potential importance of ncRNAs in the adverse effects PAE on brain development and suggest that they could be targets for novel therapeutics (Mahnke et al., 2018).

CircRNAs are a novel category of non-coding RNAs that are characterized by their unique circular secondary structure. CircRNAs are created by the back-splicing of a single-stranded linear transcript whose ends are covalently bound and are known to be much more stable than mRNAs, with a half-life time of days to a week (Jeck et al., 2013; Memczak et al., 2013; You et al., 2015; Enuka et al., 2016). Recent studies have shown that circRNAs are partially conserved between species with most highly-expressed and evolutionary-conserved circRNAs derived from exonic sequences (Lukiw, 2013; You et al., 2015; Gokool et al., 2020; Mehta et al., 2020). CircRNAs can be preferentially derived from genes that code for synaptic proteins and tend to be particularly responsive during both important stages of prenatal and postnatal brain development (Szabo et al., 2015; Dang et al., 2016), suggesting that circRNAs might serve as critical regulators of brain development and function (Rybak-Wolf et al., 2015; You et al., 2015). Recent studies have shown altered profile of expression of circRNAs in various psychiatric (Zimmerman et al., 2020) and neurodevelopmental disorders, as well as cocaine addiction (Bu et al., 2019) and Alzheimer’s disease (Dube et al., 2019). Lastly, despite the fact that previous studies have highlighted the crucial role of circRNAs in embryonic and fetal development (Venø et al., 2015; Suenkel et al., 2020), not much is known about their implications in neurodevelopmental disorders such as FASD.

Using circRNA profiling and RNA seq we previously uncovered sex-specific changes in whole brain circRNA but not mRNA expression as a result of PAE and we showed that differentially expressed by PAE circRNAs were preferentially derived from genes with important roles in brain development and function (Paudel et al., 2020). However, little is known about the potential involvement of circRNAs in the long term influence of prenatal alcohol exposure (PAE) in the brain and their relevance for Fetal Alcohol Spectrum Disorders (FASD). We have previously demonstrated that *circHomer1* is an adult brain-enriched circRNA known to be dysregulated in psychiatric disorders and able to influence synaptic gene expression and cognitive function (You et al., 2015; Zimmerman et al., 2020; Hafez et al., 2022). Focusing on factors that can control *circHomer1* biogenesis, we have reported that the RNA-binding protein (RBP) eukaryotic initiation factor 4A-III (EIF4A3), a member of the exon junction complex important for brain development and synaptic function (Giorgi et al., 2007; Mao et al., 2016), is able to positively regulate *circHomer1* expression. A previous study suggested that EIF4A3 activity can be inhibited *via* its binding to *H19* (Han et al., 2016), an imprinted embryonic brain-enriched lncRNA previously shown to be epigenetically altered following PAE and linked to FASD-associated changes in head circumference (Marjonen et al., 2017, 2018; Zhong et al., 2021; Bestry et al., 2022).

Here, we provide strong evidence that *circHomer1*, is significantly down-regulated in the male frontal cortex and hippocampus of mice subjected to modest PAE. Our data further suggest that the expression of *H19* is significantly up-regulated in the frontal cortex of male PAE mice with opposing changes in the developmental- and brain region specific- expression to that of *circHomer1*. Importantly, knockdown of *H19* in human glioblastoma cell lines resulted in a robust up-regulation of *circHomer1* but not linear *HOMER1* mRNA. Taken together, our work uncovers notable sex- and brain region-specific alterations in circRNA and lncRNA expression following PAE and introduces novel mechanistic insights with potential relevance to FASD.

## Materials and methods

### Moderate prenatal alcohol exposure (PAE)

WT C57BL/6J mice derived from Jackson Laboratory were maintained on a reverse 12 h dark/light schedule (lights on at 8:00 p.m.) in single-housed cages. We utilized a well-validated moderate PAE paradigm (Brady et al., 2012; Paudel et al., 2020) that involves giving adult female mice access to either to a solution of either 10% (w/v) ethanol and 0.066% (w/v) saccharin or 0.066% (w/v) saccharin (control) for 4 h per day. After ensuring consistent drinking, mice were given access to these solutions during mating and for the whole duration of their pregnancy. Some dams were euthanized at the end of their pregnancy and whole brain samples were extracted from embryonic day 18 (E18) male and female pups and stored in a −80°C freezer (at least 4 SAC and 4 PAE litter groups were used). For adult tissue harvesting, male and female pups were left to grow till adulthood (P80-90) without any intervention and no access to ethanol, then were euthanized, and various brain regions (frontal cortex, hippocampus, occipital cortex, cerebellum) were extracted *via* micro dissection (at least 4 SAC and 4 PAE litter groups were used). For E18 brain extractions dams was euthanized by decapitation and the body was laid on ice. The uterine horn was rapidly removed and placed on a piece of glass on top of the ice. Each embryo was removed, decapitated and the brain isolated. All adult males were euthanized by CO2 asphyxiation. All procedures were approved by the University of New Mexico Health Sciences Center Institutional Animal Care and Use Committee.

### RNA extraction and mRNA/circRNA quantification

RNA was isolated using the miRNeasy RNA isolation kit (Qiagen, Hilden, Germany) following the manufacturer’s supplied protocol. RNA quality as well as concentration of isolated total RNA was assayed through Nanodrop 2000 spectrophotometer and Qubit 3 (Thermo Fisher Scientific, Waltham, Massachusetts, USA), with all samples passing the quality control measurements (A260/230 and A260/280). Reverse transcription of total RNA (100–500 nanograms depending on the Nanodrop concentration values) was carried out using the SuperScript IV First-Strand Synthesis System (Thermo Fisher Scientific, Waltham, MA, USA) with oligo-dT for linear mRNAs and random hexamers for circRNA detection. Quantitative RT-PCR was done using either PowerUp SYBR Green Master Mix (Thermo Fisher Scientific) along with custom designed, validated, and sequence-verified circRNA and mRNA primers or TaqMan Gene Expression Assays (Thermo Fisher Scientific) for mRNA detection. All circRNA qRT-PCR products were run on an agarose gel and sequence validated. At the end of each qPCR amplifications plots and melt curves (ΔRn vs. cycle per well) were automatically calculated by Quant Studio 7 Flex. *18S rRNA* was used as a normalizer for mRNA and circRNA expression levels. For mRNA qRT-PCR quantification the following formula was used: Relative value = A^Ct^18^*^SrRNA^*/A^Ct*^mRNA^*, where A = 10^ (−1/primer slope). For circRNA qRT-PCR quantification the following formula was used: Relative value = A^Ct^18^*^S rRNA^*/A^Ct*^circRNA^*, where A = 10^ (−1/primer slope) (Zimmerman et al., 2020; Hafez et al., 2022). All primers used in this study are shown in Supplementary Table 1.

### SH-SY5y Human neuroblastoma cell *and H19* shRNA mediated KD

SH-SY5Y epithelial human neuroblastoma cell line was purchased from ATCC CRL-2266™. SY-5 can differentiate under certain circumstances. To that end, cells were fed for 5 days using Neurobasal Plus, 1XB27 Plus, 5% Pen/Strep ∼ ThermoFisher Scientific. The neuronal-like morphology was observed under the microscope and by measuring the levels of beta-3 tubulin (ab18207; Abcam, Cambridge, United Kingdom), a neuronal marker *via* immunohistochemistry as done before (Zimmerman et al., 2020). After differentiation, SY-5 cells were plated in a 24 well plate at passage #7 at a concentration of 100,000 cells per well. 48 h later they were transfected with the shRNA clone for the gene of interest, *H19*, and a non-target shRNA clone. Transfections were performed using Lipofectamine™ 3000/P3000 reagent (500 ng DNA, 1 μl Lipofectamine and 1 μl P3000 reagent per well). 48 h following the transfection, the differentiated SH-SY5Y cells were subjected to RNA extraction and then qRT-PCR to assay overall changes in *circHomer1* expression. For the *H19* KD experiments, clones were purchased from OriGene Technologies, Inc. The product datasheet used for knocking down *H19* levels was the TL318197 *H19* Human shRNA Plasmid Kit (Locus ID 283120) including *H19*–Human, 4 unique 29mer shRNA constructs and a non-effective 29-mer scrambled shRNA cassette in pGFP-C-shLenti Vector, TR30021 (Non-effective control sequence: 5′ GCACTACCAGAGCTAACTCAGATAGTACT 3′). Regarding the TL318197 *H19* Human shRNA Plasmid Kit (Locus ID 283120) the clones that were able to KD the *H19* levels were the ones with the tube ID: TL318197C and TL318197D.

## Statistical analysis

Normalized values were divided to the mean of each Control group and the relative to control ratios were plotted as means ± S.E.M. using GraphPad Prism and after removing up to 2 outliers using Roots test (Graphpad Software, La Jolla, CA, USA). For the normality and log normality of the data sets, the following tests were conducted: Anderson-Darling, D’Agostino and Pearson and Shapiro–Wilk test. Due to the fact that the vast majority of datasets passed the tests for normal Gaussian distribution, one sample *t*-test was conducted for comparing two groups, while in the few cases where data showed a non-parametric distribution the Wilcoxon Signed Rank test was used instead. For comparisons involving more than two groups, a one-way Analysis of Variance (ANOVA) with Tukey’s (comparison between all groups) or Dunnett’s (comparisons vs. a single control group) for multiple comparisons for samples with normal distribution of data and Kruskal–Wallis test with Dunn’s *post-hoc* correction for multiple comparisons was used for samples that did not display normal distribution. For comparisons comparing the effects of both treatment and sex, a two-way ANOVA with Šídák’s multiple comparisons test was used. Outliers were identified based on Rout’s test with Q = 1%. Spearman correlation coefficients and two-tailed *p*-values were calculated. Even though datasets were sampled from a Gaussian distribution, Spearman correlation was used due to the monotonic relationship of the variables and due to the more robust nature of this test for any outliers of the data set.

## Results

### Reductions in *circHomer1* in a mouse model of fetal alcohol spectrum disorder are sex- and brain-region specific

**FIGURE 1:**
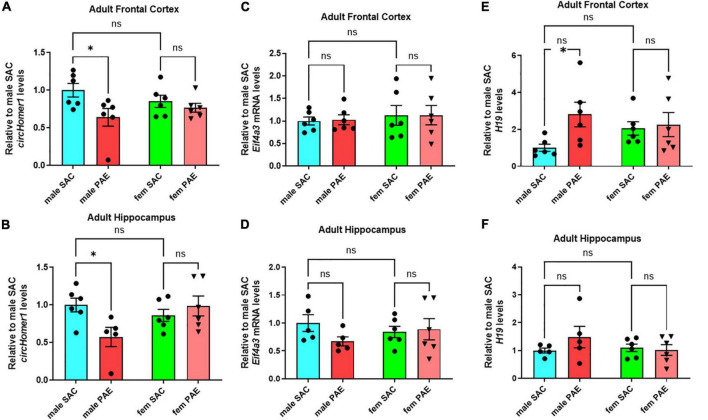
Expression of *circhomer1*, *Eif4a3*, and *H19* in the male and female adult frontal cortex and hippocampus. **(A,B)** Mean ± SEM *circHomer1* levels (normalized to *18S rRNA* and further normalized to the mean of Control male SAC *circHomer1* levels) in adult male and female (fem) PAE and SAC adult frontal cortex **(A)** and hippocampus **(B)**. For **(A)**: *p* = 0.0242, *t* = 2.838, df = 20 for male PAE vs. male SAC and *p* = 0.7539, *t* = 0.6807, df = 20 for female PAE vs. female SAC. For **(B)**: *p* = 0.0160, *t* = 2.962, df = 19 for male PAE vs. male SAC and *p* = 0.7461, *t* = 0.6940, df = 19 for female PAE vs. female SAC. **(C,D)** Mean ± SEM relative to male SAC *Eif4a3* mRNA levels (normalized to *18S rRNA*) in adult male and female (fem) PAE and SAC frontal cortex **(C)** and hippocampus **(D)**. For (C): *p* = 0.9909, *t* = 0.1213, df = 20 for male PAE vs. male SAC and *p* = 0.995, *t* = 0.02928, df = 20 for female PAE vs. female SAC. For **(D)**: *p* = 0.2506, *t* = 1.568, df = 18 for male PAE vs. male SAC and *p* = 0.9575, *t* = 0.2647, df = 20 for female PAE vs. female SAC. **(E,F)** Mean ± SEM relative to male SAC *H19* levels (normalized to *18S rRNA*) in adult male and female (fem) PAE and SAC frontal cortex **(E)** and hippocampus **(F)**. For **(E)**: *p* = 0.0250, *t* = 2.741, df = 20 for male PAE vs. male SAC and *p* = 0.9504, *t* = 0.2868, df = 20 for female PAE vs. female SAC. For **(F)**: *p* = 0.2930, *t* = 1.469, df = 18 for male PAE vs. male SAC and *p* = 0.9569, *t* = 0.2670, df = 18 for female PAE vs. female SAC. In **(A–F)**: ^*^p < 0.05 based on two-way ANOVA corrected with Šídák’s multiple comparisons test. Individual biological replicates are shown in each graph.

Using a well-established mouse model of moderate FASD (Brady et al., 2012; Paudel et al., 2020) in which adult female mice were given either a solution of 10% (w/v) ethanol and 0.066% (w/v) saccharin or just 0.066% (w/v) saccharin (SAC; control mice) for a total period of 4 h per day during mating and during the whole duration of their pregnancy, we had previously shown that prenatal ethanol exposure (PAE) can disturb the expression of circRNAs in a sex-specific manner in fetal brain (Paudel et al., 2020). In order to determine the long-term effects of PAE on postnatal brain circRNA expression, we used the same model of moderate PAE, but allowed SAC and PAE offspring to grow without any treatment into adulthood (P80-P90) and euthanized them to extract various brain regions of interest (frontal cortex, hippocampus, occipital cortex, cerebellum). We decided to focus on *circHomer1*, a brain-enriched circRNA previously shown to be associated with psychiatric and neurological disease, and known to regulate synaptic gene expression and cognitive function (Zimmerman et al., 2020; Hafez et al., 2022). In addition to the fact that *circHomer1* is a well-studied circRNA with important implications for neuronal function and behavior (Zimmerman et al., 2020; Hafez et al., 2022), we have previously demonstrated that down-regulation of *circHomer1* can impact the synaptic localization of numerous synaptic plasticity genes, including genes known to be implicated in FASD (Zimmerman et al., 2020; Hafez et al., 2022). Focusing on the changes in *circHomer1* levels in adult brain as a result of prenatal exposure to alcohol, we found that *circHomer1* was specifically reduced in male but not female adult frontal cortex and hippocampus (Figures 1A, B; two-way ANOVA with *post-hoc* Šídák’s multiple comparisons test). However, no changes in *circHomer1* levels were observed in adult occipital cortex and cerebellum due to PAE (Supplementary Figures 1A, B). These data suggest that *circHomer1* is down-regulated in a brain region- and sex-specific manner in adult mouse brain as a result of prenatal exposure to alcohol.

### Increased *H19* expression in male frontal cortex of mice prenatally-exposed to alcohol

We have previously shown that Eukaryotic initiation factor 4A-III (EIF4A3), an RNA-Binding protein (RBP) and component of the exon-junction complex known to be associated with circRNA biogenesis, can promote the expression of *circHomer1* (Hafez et al., 2022). Quantification of *Eif4a3* mRNA showed no significant changes in *Eif4a3* mRNA levels in response to PAE in male frontal cortex and hippocampus or the rest of the examined brain regions (Figures 1C, D and Supplementary Figures 1C, D; a 30% decrease in *Eif4a3* mRNA levels was found in male PAE hippocampus, but it did not reach statistical significance with ANOVA and *post-hoc* Šídák’s multiple comparisons test). Thus, it is not likely, that changes in *Eif4a3* mRNA could be behind the significant down-regulation of *circHomer1* in adult male frontal cortex.

In order to identify additional genes that could modulate EIF4A3-mediated *circHomer1* biogenesis, we focused on the imprinted lncRNA *H19*, which has been previously shown to bind to EIF4A3 and obstruct its recruitment to downstream RNA targets (Han et al., 2016). Given that *H19* could potentially inhibit EIF4A3-mediated *circHomer1* biogenesis, we hypothesized that PAE-induced changes into *H19* expression might be opposite to what was observed for *circHomer1*. Focusing on changes in adult brain as a result of PAE, we found a more than 2.8-fold up-regulation in *H19* expression in male PAE adult frontal cortex (Figure 1E; based on ANOVA with *post-hoc* Šídák’s multiple comparisons test). However, changes in *H19* expression in other adult brain regions in response to PAE were unremarkable (Figure 1F and Supplementary Figures 1E, F; a 48% non-significant increase in *H19* levels was seen in male PAE hippocampus). Of note, the expression of *Gas5* lncRNA, another lncRNA with potential relevance to PAE, also showed a modest up-regulation only in male PAE frontal cortex (Supplementary Figures 2A–D). We conclude that reduced *circHomer1* levels in adult male frontal cortex as a result of developmental exposure to alcohol are accompanied by significant increases in *H19* lncRNA expression.

### Opposing brain region- and developmental-specific changes in *H19* and *circHomer1* expression in mouse brain

**FIGURE 2:**
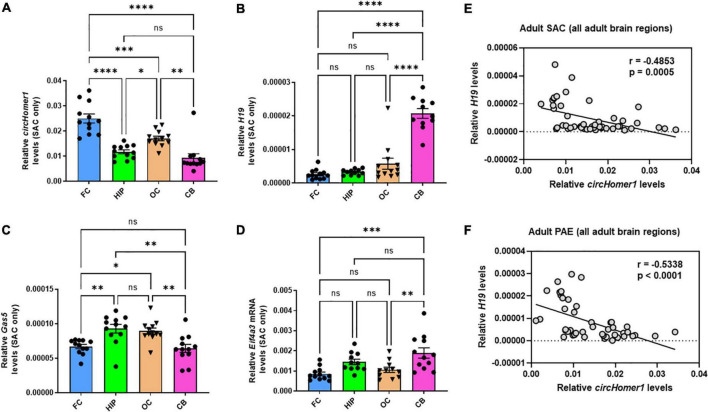
Brain region-specificity of *circhomer1*, *H19*, *Gas5*, and *Eif4a3* mRNA in four different adult brain regions. **(A–D)** Mean ± SEM relative *circHomer1* **(A)**, *H19* **(B)**, and Gas5 **(C)**, and *Eif4a3* mRNA **(D)** levels (normalized to 18S rRNA) in adult SAC frontal cortex (FC), hippocampus (HIP), occipital (OC), and cerebellum (CB). For *circHomer1* **(A)**: FC vs. HIP, *p* < 0.0001, q = 9.691, df = 44. For FC vs. OC, *p* = 0.001, q = 5.804, df = 44. For FC vs. CB, *p* < 0.0001, q = 11.49, df = 44. For HIP vs. OC, *p* = 0.0414, q = 3.888, df = 44. For HIP vs. CB, *p* = 0.5840, q = 1.802, df = 44. For OC vs. CB, *p* = 0.0012, q = 5.689, df = 44. For *H19* **(B)**: FC vs. HIP, *p* < 0.0001, q = 9.691, df = 44. For FC vs. OC, *p* = 0.001, q = 5.804, df = 44. For FC vs. CB, *p* < 0.0001, q = 11.49, df = 44. For HIP vs. OC, *p* = 0.0414, q = 3.888, df = 44. For HIP vs. CB, *p* = 0.5840, q = 1.802, df = 44. For OC vs. CB, *p* = 0.0012, q = 5.689, df = 44. For *H19* **(B)**: For FC vs. HIP, *p* = 0.9743, q = 0.5976, df = 42. For FC vs. OC, *p* = 0.2030, q = 2.833, df = 42. For FC vs. CB, *p* < 0.0001, q = 15.95, df = 42. For HIP vs. OC, *p* = 0.4252, q = 2.173, df = 42. For HIP vs. CB, *p* < 0.0001, q = 15.03, df = 42. For OC vs. CB, *p* < 0.0001, q = 13.18, df = 42. For *Gas5* **(C)**: For FC vs. HIP, *p* = 0.0037, q = 5.162, df = 44. For FC vs. OC, *p* = 0.0143, q = 4.479, df = 44. For FC vs. CB, *p* = 0.9780, q = 0.5662, df = 44. For HIP vs. OC, *p* = 0.9625, q = 0.6827, df = 44. For HIP vs. CB, *p* = 0.0011, q = 5.728, df = 44. For OC vs. CB, *p* = 0.0047, q = 5.046, df = 44. For *Eif4a3* **(D)**: For FC vs. HIP, *p* = 0.0722, q = 3.556, df = 42. For FC vs. OC, *p* = 0.8258, q = 1.215, df = 42. For FC vs. CB, *p* = 0.0003, q = 6.355, df = 42. For HIP vs. OC, *p* = 0.3784, q = 2.292, df = 42. For HIP vs. CB, *p* = 0.2518, q = 2.659, df = 42. For OC vs. CB, *p* = 0.0054, q = 5.000, df = 42. For **(A–D)** ^*^p < 0.05, ^**^p < 0.01, ^***^p < 0.001, ^****^p < 0.0001, based on one way ANOVA with *post-hoc* Tukey’s multiple comparisons test. **(E,F)** Correlation between relative *circHomer1* and *H19* in all four brain regions of adult SAC **(E)** and PAE **(F)** mice. Spearman coefficient and two-tailed *p*-value is shown in the graph. Individual biological replicates are shown in each graph.

Given the opposing changes in *circHomer1* and *H19* expression in the frontal cortex of adult male PAE mice, we decided to investigate whether the expression of *circHomer1* and *H19* could be displaying differential developmental- or brain region- specific changes in control mouse brains. Looking at the overall expression of *circHomer1* in frontal cortex, hippocampus, occipital cortex, and cerebellum of control SAC mice, we noticed that it was significantly enriched in adult frontal cortex (Figure 2A), a result which is consistent with previous findings in untreated mouse brain (Zimmerman et al., 2020). Expression of *circHomer1* was intermediate in adult occipital cortex and hippocampus, with the lowest levels detected in adult SAC cerebellum (Figure 2A). Previous studies have shown *H19* is a prenatal stage-enriched lncRNA important for embryogenesis (Poirier et al., 1991; Gabory et al., 2009; Li et al., 2019) and previously implicated in FASD (Marjonen et al., 2017, 2018; Bestry et al., 2022). However, its expression was still detectable in the adult brain in our control SAC brain samples, with higher levels in the cerebellum and lower expression in the adult frontal cortex (Figure 2B), which is the opposite of what was observed for *circHomer1*. On the other hand, Gas5 was expressed in comparable levels in frontal cortex and cerebellum, with a modest enrichment in hippocampus and occipital cortex (Figure 2C). Lastly, measuring the expression of *Eif4a3* mRNA, we found that it did not exhibit notable brain region-specific changes in control SAC adult brain, with an exception of a slightly higher expression in the cerebellum (Figure 2D). Comparing the brain region-specific expression of *circHomer1* to *H19* (Figure 2E) and *Gas5*, we found a significant inverse correlation between *circHomer1* and *H19* (Figure 2E), but not *circHomer1* and *Gas5* (*r* = −0.1218, *p* = 0.4097) in adult SAC brains. Of note, a negative correlation was also observed between *circHomer1* and *Eif4a3* across SAC brain regions (*r* = −0.4260, *p* = 0.0032). Interestingly, the negative correlation between *H19* and *circHomer1* between adult brain regions was also observed in PAE animals (Figure 2F).

**FIGURE 3:**
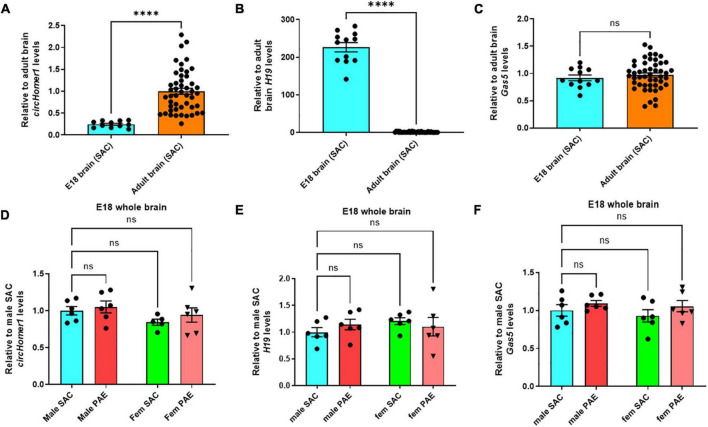
Developmental-specificity of *circhomer1*, *H19*, *Gas5* in control brain. **(A–C)** Mean ± SEM relative to adult brain *circHomer1* **(A)**, *H19* **(B)**, and *Gas5* **(C)** levels (normalized to 18S rRNA) in E18 whole brain and adult SAC brain (data from all four brain regions are included). For *circHomer1* **(A)**: *p* < 0.0001, *t* = 33.77, df = 10. For *H19* **(B)**: *p* < 0.0001, *t* = 18.36, df = 11. For *Gas5* **(C)**: *p* = 0.15020, *t* = 1.547, df = 11. For **(A–C)**: ^****^p < 0.0001 based on two-tailed one-sample *t*-test. Number of biological replicates shown in the graph. **(D–F)** Mean ± SEM relative to male SAC *circHomer1* **(D)**, *H19* **(E)**, and *Gas5* **(F)** levels (normalized to 18S rRNA) in E18 male and female (fem) PAE and SAC whole brain. For *circHomer1* **(D)**: *p* = 0.8558, *t* = 0.5038, df = 19 for male PAE vs. male SAC, and *p* = 0.6115, *t* = 0.9052, df = 19 for female PAE vs. female SAC. For *H19* **(E)**: *p* = 0.6151, *t* = 0.8985, df = 20 for male PAE vs. male SAC and *p* = 0.7705, *t* = 0.6535, df = 20 for female PAE vs. female SAC. For *Gas5* **(F)**: *p* = 0.5839, *t* = 0.9471, df = 20 for male PAE vs. male SAC and *p* = 0.3797, *t* = 1.288, df = 20 for female PAE vs. female SAC. For **(D–F)**, a two-way ANOVA corrected with Šídák’s multiple comparisons test. Individual biological replicates are shown in each graph.

We then compared their expression of *circHomer1* and *H19* and *Gas5* in adult frontal cortex, hippocampus, occipital cortex, and cerebellum of control SAC mice vs. E18 prenatal whole brain from SAC mice, in order to determine their developmental-specific expression in control animals. We found that *circHomer1* levels in control SAC mice were very low in the E18 brain, but robustly increased in the adult brain, with the highest expression in adult frontal cortex (Figure 3A), which is in accordance with previous findings in WT untreated mice (Zimmerman et al., 2020). Given that *H19* can inhibit EIF4A3-mediated *circHomer1* biogenesis, we hypothesized that developmental and PAE-induced changes into *H19* expression could potentially explain the observed changes in *circHomer1* expression during brain development. Our results showed that *H19* is significantly enriched in the prenatal brain of SAC control mice in comparison to adult brain (Figure 3B; close to 200-fold more *H19* found in prenatal brain), which is in accordance to previous literature suggesting that *H19* is a prenatal stage-enriched lncRNA important for embryogenesis (Poirier et al., 1991; Gabory et al., 2009; Li et al., 2019; Zhong et al., 2021). However, no changes were observed in Gas5 expression between E18 whole brain and adult brain (Figure 3C). Of note, looking at the PAE-induced changes in prenatal E18 whole brain, we found no significant effects on *circHomer1*, *H19*, *or Gas5* expression (Figures 3D–F). We conclude that changes in *circHomer1* expression during normal brain development and within different brain regions in the adult brain are inversely associated with *H19* expression and that PAE does not significantly impact their expression in E18 prenatal brain.

### H19 can inhibit *circHomer1* biogenesis

**FIGURE 4:**
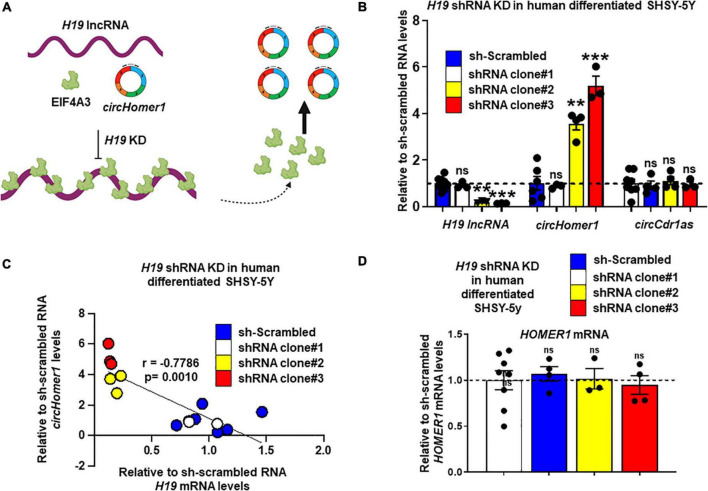
*H19* knockdown increases *circHomer1* expression. **(A)** Proposed diagram depicting the hypothesized mechanism for *H19*/EIF4A3 regulation of *circHomer1* biogenesis. *H19* can bind and sequester EIF4A3, thus inhibiting EIF4A3-mediated *circHomer1* biogenesis. **(B)** Relative *H19*, *circHomer1*, and *circCDR1as* levels (based on qRT-PCR and normalized to *18s rRNA*) 48 h after shRNA-mediated knockdown of *H19* in human differentiated SH-SY5Y cells. Mean ± SEM. **p < 0.01, ***p < 0.001, two-tailed one sample *t*-test relative to shRNA scrambled. For *H19* for clone 2: *p* = 0.0011, *t* = 30.26, df = 2. For *H19* for clone 3: *p* < 0.0001, *t* = 116.7, df = 2. For *circHomer1* for clone 2: *p* = 0.0024, *t* = 9.612, df = 3. For *circHomer1* for clone 3: *p* = 0.0008, *t* = 14.09, df = 3. **(C)** Correlation between relative sh-scrambled H19 and *circHomer1* levels following *H19* shRNA Knockdown in human differentiated SHSY-5Y. Spearman coefficient and two-tailed *p*-value is shown in the graph. **(D)** Relative to sh-scrambled *HOMER1* mRNA levels (based on qRT-PCR and normalized to 18s rRNA) 48 h after shRNA-mediated knockdown of *H19* in human differentiated SH-SY5Y cells. Mean ± SEM. For clone 2: *p* = 0.8968, *t* = 0.1467, df = 2. For clone 3: *p* = 0.6432, *t* = 0.5132, df = 3. Individual biological replicates are shown in each graph.

Based on *in silico* analysis of RBP binding sites (Li et al., 2014), *H19* is predicted to have 17 different binding sites for EIF4A3. We hypothesized that direct binding of multiple EIF4A3 proteins on *H19*, would reduce the availability of EIF4A3 to promote *circHomer1* biogenesis (Figure 4A). In order to test this hypothesis, we utilized shRNA-mediated KD of *H19* in human differentiated SHSY-5Y cells. We used three different shRNAs against *H19* and extracted RNA for *circHomer1* and *H19* measurements *via* qRT-PCR after 2 days of shRNA treatment. Our results suggested that shRNA clones 2 and 3 resulted in a robust reduction in *H19*, whereas shRNA clone 1 was not efficient in knocking down *H19* (Figure 4B). Remarkably, *circHomer1* levels were unchanged following treatment with shRNA clone 1 but were robustly up-regulated after *H19* knockdown with shRNA clones 2 and 3 (Figure 4B). This effect was specific to *circHomer1* since no changes were seen in *circCDR1as* following *H19* knockdown (Figure 4B). Interestingly, relative *circHomer1* expression was found to be significantly inversely associated with *H19* levels (Figure 4C), further suggesting that the level of *H19* knockdown was inversely proportional to the up-regulation observed in *circHomer1* expression. However, no changes were seen in overall *HOMER1* mRNA levels in this experiment (Figure 4D).

## Conclusion

Numerous studies have suggested the importance of circRNAs for brain development and function and their relevance for neurodevelopmental and psychiatric disorders (Lukiw, 2013; Mehta et al., 2020; Zimmerman et al., 2020; Li M. L. et al., 2021; Hafez et al., 2022). Our previous work uncovered circRNAs associated with neurogenesis and neuronal development that are altered in prenatal brain in a sex-specific manner as a result of PAE (Paudel et al., 2020). Moreover, additional research has suggested that circRNAs could be associated with the biological mechanisms underlying alcohol addiction or could serve as biomarkers of alcohol dependence (Liu et al., 2021; Vornholt et al., 2021). However, the role of circRNAs in the long-term effects of PAE and the mechanisms that could underlie PAE-associated changes in circRNA expression have not being adequately explored. Here we show evidence that *circHomer1*, an abundantly expressed circRNA in adult brain that has been previously linked to cognitive function and psychiatric disorders (Zimmerman et al., 2020; Hafez et al., 2022), is significantly down-regulated in the frontal cortex and hippocampus of adult male but not female mice prenatally exposed to alcohol. Moreover, we demonstrate that *H19*, an imprinted lncRNA known to inhibit the function of an RBP capable of promoting *circHomer1* biogenesis, is significantly up-regulated in male adult frontal cortex and displays a developmental- and brain region-specific expression that is opposite to what is observed for *circHomer1*. Lastly, we show that knockdown of H19 results in a significant up-regulation of *circHomer1* in human glioblastoma cell lines. Taken together our results, uncover a novel antagonistic interaction between a circRNA and a lncRNA with potential relevance to brain development and FASD.

Previous work from our lab and others have suggested that *circHomer1* is an activity-dependent, postnatal brain-enriched circRNA, that can significantly affect synaptic gene expression, *Homer1* mRNA isoform synaptic localization, neuronal activity, and frontal cortex-mediated cognitive flexibility (Giorgi et al., 2007; Venø et al., 2015; Zimmerman et al., 2022). Moreover, changes in *circHomer1* expression have been reported in numerous psychiatric and neurological disorders (Dube et al., 2019; Zimmerman et al., 2020; Hafez et al., 2022). We recently showed that EIF4A3, an RBP associated with the exon junction complex, binds to *circHomer1* and is a potent positive regulator for its biogenesis (Hafez et al., 2022). We also demonstrated that both *circHomer1* and *Eif4a3* are experience-dependent in the frontal cortex and appear to be positively correlated in mouse and human frontal cortex (Hafez et al., 2022). However, in this current study we found that PAE induces a significant down-regulation in the expression of *circHomer1* in adult male frontal cortex without any changes in *Eif4a3* mRNA expression; thus making it unlikely that such changes in *circHomer1* levels are a result of a transcriptional increase in *Eif4a3* expression. Looking at potential regulators of EIF4A3 activity, however, we were able to find a robust up-regulation in male PAE frontal cortex in the expression of *H19*, a lncRNA that we also found to have a strong inhibitory effect in *circHomer1* expression. Given the previous report that *H19* can bind to EIF4A3 protein and sequester it from binding to its RNA targets, it is tempting to hypothesize, that an up-regulation in *H19* levels could result in reduced EIF4A3-mediated *circHomer1* synthesis. The fact that the developmental and brain region-specific expression of *circHomer1* and *H19* appeared to be inversely associated further supports the potential of an inhibitory interaction between these important ncRNAs. Furthermore, given the robust developmental down-regulation of *H19* in the postnatal brain, it is tempting to hypothesize that PAE could delay or reduce this normal developmental decrease in *H19* expression *via* potential epigenetic mechanisms such as DNA methylation. This in turn could result in relatively higher levels of *H19*, which can in turn down-regulate *circHomer1* expression in adult animals following PAE. However, additional research will be needed to further explore such a potential mechanism, especially given the many reported mechanisms of action for H19 (Bestry et al., 2022). Moreover, the modest increases in *H19* and reductions in *Eif4a3* mRNA observed in adult male hippocampus as a result of PAE were not significant, thus allowing for other potential mechanisms to explain the observed changes in *circHomer1* in this brain region.

One important limitation of our work is that our data were limited to E18 and adult stages of development, so that we were not able to properly access the exact developmental trajectories in gene expression that could have resulted from prenatal alcohol exposure. Moreover, our experiment using knockdown of *H19* was conducted in human neuroblastoma cell lines and was not replicated in mouse neuronal cultures or *in vivo.* On a similar note, our measurements of *H19* levels in adult mouse brains displayed high overall variability due to the low expression of this lncRNA in adult stages of development. It is worth mentioning that cessation of alcohol exposure in our mouse model of FASD was after birth, since dams were exposed to alcohol during breeding and gestation. As a result, do not believe that our observed effects in gene expression in adult mouse brains are likely to be a result of withdrawal given the prolonged time from cessation of alcohol exposure. Furthermore, our previous work identified numerous genes involved in synaptic plasticity with potential relevance to FASD to be up-regulated within synaptosomes following *in vivo* knockdown of *circHomer1* in the frontal cortex (Zimmerman et al., 2020). Among these genes, were Glutamate [NMDA] receptor subunit epsilon-2 (*Glun2*), Fragile X Messenger Ribonucleoprotein 1 (*Fmr1*), Sodium channel protein type 1 subunit alpha (*Scn1a*), Neurexin 1 (*Nrxn1*), and cAMP response element-binding protein (*Creb*) (Zimmerman et al., 2020). Therefore, future work is needed to identify whether PAE-mediated changes in *circHomer1* in adult brain could be involved in the dysregulation of synaptic gene expression.

Previous work has uncovered sex-specific effect of PAE in brain and other tissues (Weinberg et al., 2008; Terasaki et al., 2016; Loke et al., 2018; Bake et al., 2021). On a similar note, our previous study revealed that circRNAs are altered in a sex-specific manner in E18 prenatal brain following PAE (Paudel et al., 2020). Interestingly, *H19* has been reported to have sex-specific expression and to be differentially affected by estrogen and androgen treatment (Sun et al., 2015; Basak et al., 2018; Ozgur and Gezer, 2020; Li T. et al., 2021). Moreover, *H19* has been previously linked with changes in head circumference in FASD and reported to be epigenetically altered in various tissues (Marjonen et al., 2017, 2018; Bestry et al., 2022). Taken together, this allows us to hypothesize that PAE could induce epigenetic changes in the *H19* locus in a sex-specific manner, which could result in opposing changes in *circHomer1* expression. However, new research is needed to potentially account for the brain region-specific effects observed in *H19 and circHomer1* expression as a result of PAE.

Regardless of the exact mechanism underlying the inhibitory effect of *H19* on *circHomer1*, it is also worth mentioning that our findings are the first to uncover such an antagonist effect between a lncRNA and a circRNA in the brain. Based on our data this inhibitory effect of *H19* on *circHomer1* expression, could potentially be important for normal brain development and maturation, as well as the control of brain region-specific expression for *circHomer1*. Given the link of *circHomer1* with additional neuropsychiatric disorders (Zimmerman et al., 2020; Hafez et al., 2022), and the increased prevalence of neuropsychiatric disturbances in patients with FASD, it would be interesting to further dissect the interaction between *H19* and *circHomer1*, and mechanistically study its relevance to brain development, function, disease.

## Data availability statement

The original contributions presented in the study are included in the article/Supplementary material, further inquiries can be directed to the corresponding author/s.

## Ethics statement

The animal study was reviewed and approved by University of New Mexico Health Sciences Center Institutional Animal Care and Use Committee.

## Author contributions

NM and GP conceived the hypothesis, designed and conducted the experiments, analyzed the data, and wrote the manuscript. AA and KC designed and conducted the experiments. All authors conducted the experiments and analyzed the data and helped with manuscript preparation.
