# Supplementary material for: Prenatal alcohol exposure results in brain region- and sex-specific changes in circHomer1 expression in adult mouse brain
Source: Front Neurosci. 2023 Feb 17;17:1087950. doi: 10.3389/fnins.2023.1087950 (PMC9983553; doi:10.3389/fnins.2023.1087950)
Supplement: Supplementary file 1 [file Data_Sheet_1.docx]

**SUPPLEMENTAL MATERIAL**

**Prenatal alcohol exposure results in brain region- and sex-specific changes in *circHomer1* expression in adult mouse brain.**

**Supplementary Figure 1: No changes in *circhomer1*, *Eif4a3*, and *H19* levels in adult male and female occipital cortex and cerebellum as a result of PAE.**

A-B) Mean ± SEM relative to male SAC *circHomer1* levels (normalized to 18S rRNA) in adult male and female (fem) PAE and SAC adult occipital cortex (A) and cerebellum (B). For A: p = 0.9984, t = 0.05146, df = 19 for male PAE vs male SAC and p > 0.9999, t = 0, df = 19 for female PAE vs female SAC. For B: p = 0.7533, t = 0.6823, df = 19 for male PAE vs male SAC and p = 0.9989, t = 0.04209, df = 19 for female PAE vs female SAC. C-D) Mean ± SEM relative to male SAC *Eif4a3* mRNA levels (normalized to 18S rRNA) in adult male and female (fem) PAE and SAC adult occipital cortex (C) and cerebellum (D). For C: p = 0.9874, t = 0.1431, df = 19 for male PAE vs male SAC and p = 0.6022, t = 0.9197, df = 19 for female PAE vs female SAC. For D: p = 0.8601, t = 0.495, df = 20 for male PAE vs male SAC and p = 0.8792, t = 0.4572, df = 20 for female PAE vs female SAC. E-F) Mean ± SEM relative to male SAC *H19* levels (normalized to 18S rRNA) in adult male and female (fem) PAE and SAC adult occipital cortex (E) and cerebellum (F). For E: p = 0.5764, t = 0.9587, df = 20 for male PAE vs male SAC and p = 0.9755, t = 0.1999, df = 20 for female PAE vs female SAC. For F: p = 0.1122, t = 2.026, df = 18 for male PAE vs male SAC and p = 0.0584, t = 2.362, df = 18 for female PAE vs female SAC. For A-F, a two-way ANOVA corrected with Šídák's multiple comparisons test was used. Individual biological replicates are shown in each graph.

**Supplementary Figure 2: Changes in *Gas5* levels in four different regions of the adult brain as a result of PAE.**

A-D) Mean ± SEM relative to male SAC *Gas5* levels (normalized to 18S rRNA) in adult male and female (fem) PAE and SAC adult frontal cortex (A), hippocampus (B), occipital cortex (C), and cerebellum (D). For A: p = 0.0391, t = 2.534, df = 20 for male PAE vs male SAC and p = 0.1821, t = 1.749, df = 20 for female PAE vs female SAC. For B: p = 0.4667, t = 1.137, df = 19 for male PAE vs male SAC and p = 0.9622, t = 0.2498, df = 19 for female PAE vs female SAC. For C: p = 0.4175, t = 1.222, df = 19 for male PAE vs male SAC and p = 0.6413, t = 0.8589, df = 19 for female PAE vs female SAC. For D: p = 0.6359, t = 0.8663, df = 20 for male PAE vs male SAC and p = 0.7970, t = 0.6090, df = 20 for female PAE vs female SAC. For A-D: *p<0.05 based on two-way ANOVA corrected with Šídák's multiple comparisons test. Individual biological replicates are shown in each graph.


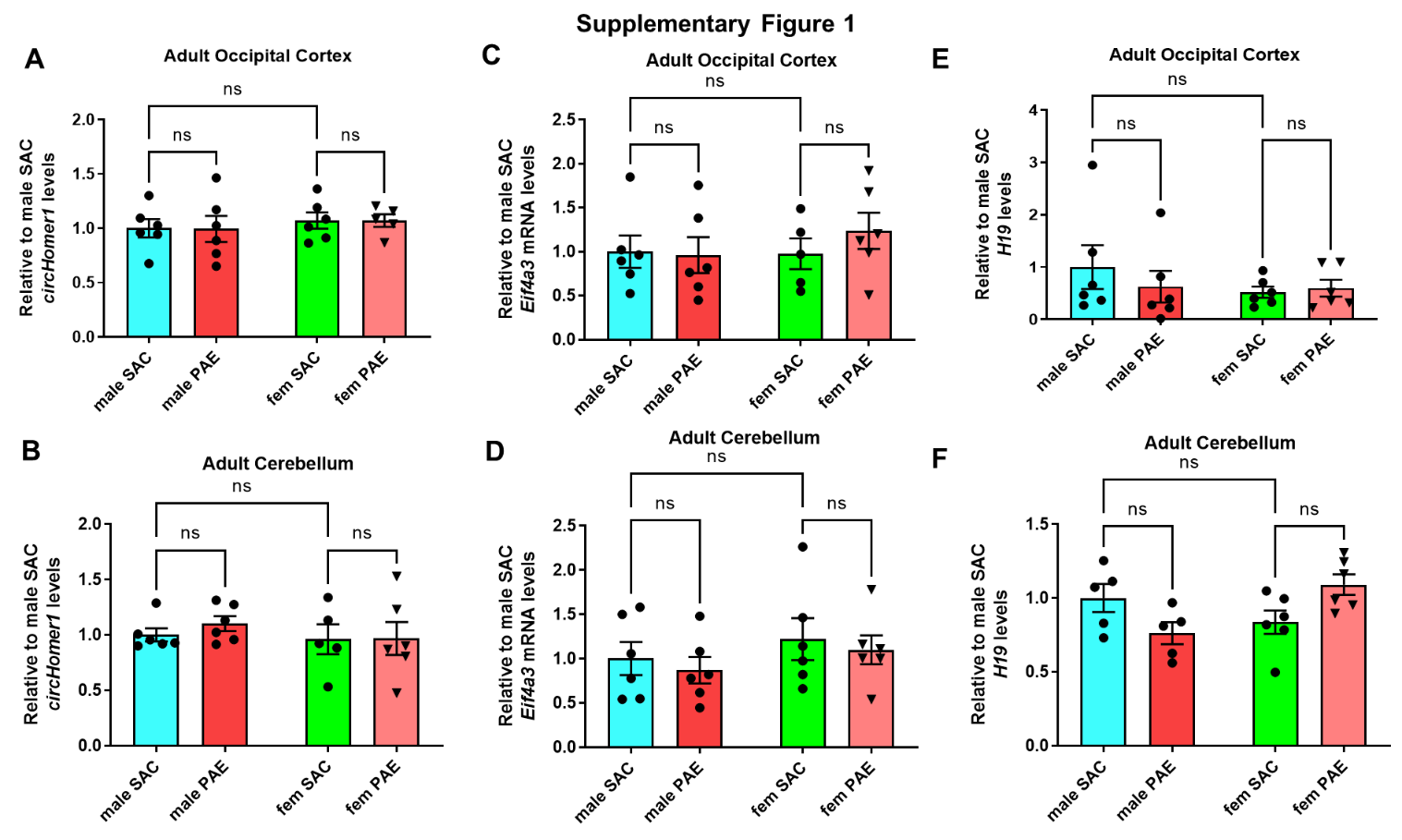


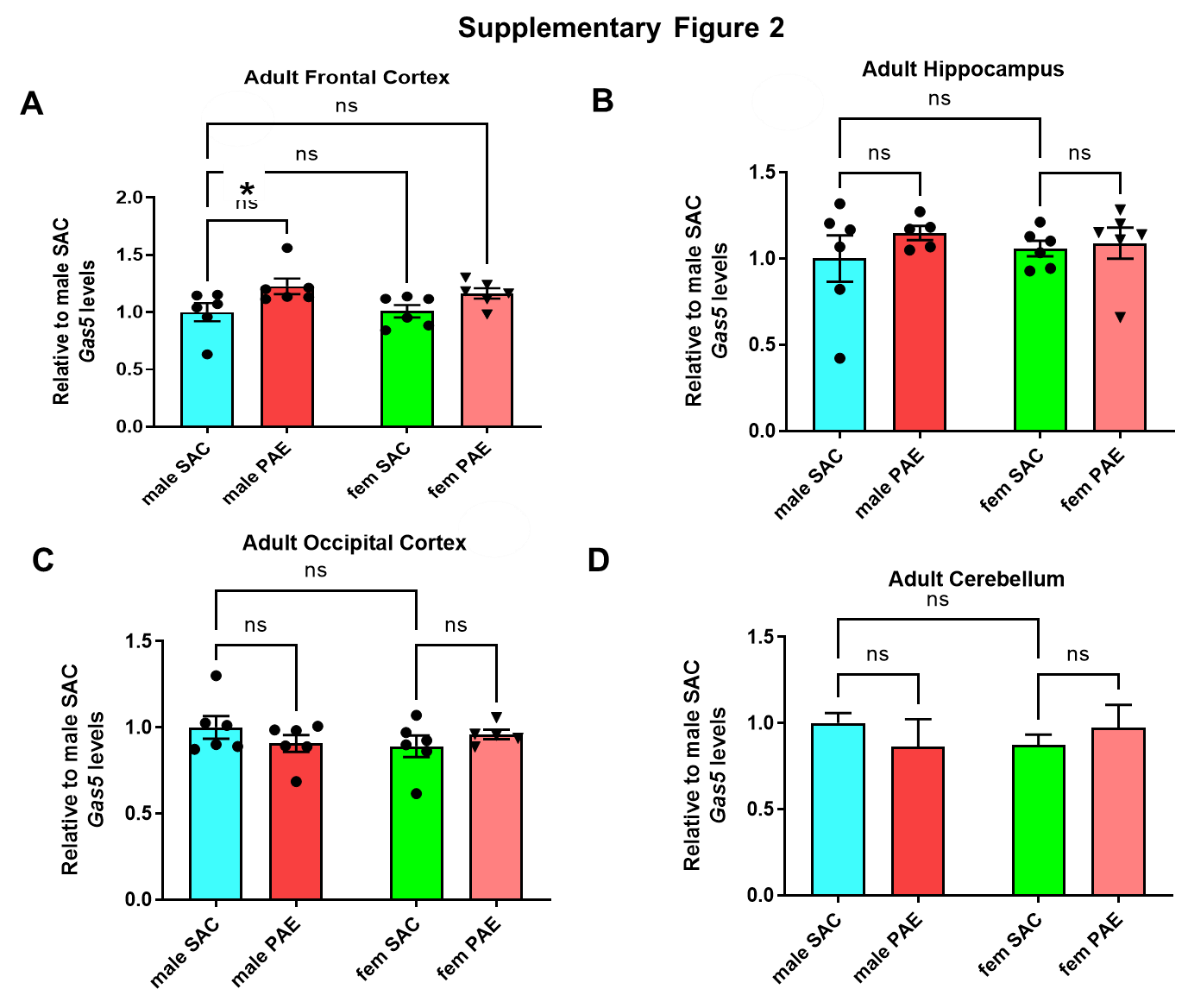


**Supplementary Table 1: List of Primers.**

| **Primer name** | **Species** | **Transcript name** | **Primer Sequence or assay ID** |
| --- | --- | --- | --- |
| *h_HOMER1all* | Human | *HOMER1* | Hs01029333_m1 |
| *h_18S_Taqman* | human | *18S rRNA* | Hs99999901_s1 |
| *h_H19_Taqman* | Human | *H19* | Hs00399294_g1 |
| *h_circHomer1-F* | Human | *circHomer1* | TCAACGGGACAGATGATGAA |
| *h_circHomer1-R* | Human | *circHomer1* | TTGTGTTTGGGTCAATTTGG |
| *h_CDR1as-F* | Human | *CDR1as* | ACGTCTCCAGTGTGCTGA |
| *h_CDR1as-R* | Human | *CDR1as* | CTTGACACAGGTGCCATC |
| *m_18S_Taqman* | mouse/rat | *18S rRNA* | Mm03928990_g1 |
| *m_Eif4a3_Taqman* | Mouse | *Eif4a3* | Mm00836350_g1 |
| *m_circHomer1-F* | Mouse | *circHomer1* | TTCCACATAGGGAGCAACC |
| *m_circHomer1-R* | Mouse | *circHomer1* | TCTTCTTTGTGTTCGGGTCA |
| *m_H19_Taqman* | Mouse | *H19* | Mm01156721_g1 |
| *m_Gas5_Taqman* | Mouse | *Gas5* | Mm00657322_g1 |
